# The Perceived Stress Scale 2&2: a two-factorial German short version of the Perceived Stress Scale

**DOI:** 10.3389/fpsyt.2023.1195986

**Published:** 2023-07-06

**Authors:** Sarah K. Schäfer, Lisa von Boros, Anja S. Göritz, Sophie Baumann, Michèle Wessa, Oliver Tüscher, Klaus Lieb, Anne Möhring

**Affiliations:** ^1^Leibniz Institute for Resilience Research, Mainz, Germany; ^2^Department for Clinical Psychology, Psychotherapy and Psychodiagnostics, Technische Universität Braunschweig, Braunschweig, Germany; ^3^Behavioral Health Technology, Augsburg University, Augsburg, Germany; ^4^Department Methods in Community Medicine, University Medicine Greifswald, Greifswald, Germany; ^5^Department of Clinical Psychology and Neuropsychology, Institute for Psychology, Johannes Gutenberg University Mainz, Mainz, Germany; ^6^Department of Psychiatry and Psychotherapy, University Medical Center of Johannes Gutenberg University, Mainz, Germany; ^7^Institute for Molecular Biology, Mainz, Germany

**Keywords:** stress, Perceived Stress Scale, assessment, short version, German, resilience, machine learning, ant-colony-optimization

## Abstract

**Background:**

Stress is among the leading causes for diseases. The assessment of subjectively perceived stress is essential for resilience research. While the Perceived Stress Scale (PSS) is a widely used questionnaire, a German short version of the scale is not yet available. In the current study, we developed such a short version using a machine learning approach for item reduction to facilitate the simultaneous optimization of multiple psychometric criteria.

**Method:**

We recruited 1,437 participants from an online panel, who completed the German long version of the PSS along with measures of mental health and resilience. An ant-colony-optimization algorithm was used to select items, taking reliability, and construct validity into account. Findings on validity were visualized by psychological network models.

**Results:**

We replicated a bifactor structure for the long version of the PSS and derived a two-factor German short version of the PSS with four items, the PSS-2&2. Its factors helplessness and self-efficacy showed differential associations with mental health indicators and resilience-related factors, with helplessness being mainly linked to mental distress.

**Conclusion:**

The valid and economic short version of the PSS lends itself to be used in future resilience research. Our findings highlight the importance of the two-factor structure of the PSS short versions and challenge the validity of commonly used one-factor models. In cases where the general stress factor is of interest, researchers should use the longer versions of the PSS that allow for the interpretation of total scores, while the PSS-2&2 allows of an economic assessment of the PSS factors helplessness and self-efficacy.

## 1. Introduction

Stress is among the leading causes for the onset and persistence of illness (1). However, the pathogenic impact of stress largely depends on the subjective perception and appraisal of internal and external stimuli. According to the transactional stress model (2), subjective stress arises from a situation being perceived as threatening and where situational demands exceed individual coping resources. In line with this notion, studies demonstrated that physiological stress is insufficient to predict subjectively perceived stress (3, 4), pointing to the key role of cognitive appraisal for perceived stress.

The most widely used instrument for the assessment of subjectively perceived stress is the Perceived Stress Scale [PSS; (5, 6)]. The PSS assesses the extent to which individuals experience everyday situations as uncontrollable, unpredictable, and overwhelming relative to their coping resources (7). In contrast to other measures, the PSS assesses stress in a general manner and not related to specific life domains or components of stress models, and is relatively economic (7, 8). The PSS has been developed as a scale comprising two factors, that is, perceived helplessness and perceived self-efficacy, although some studies found a single-factor structure (9) and, more recently, bifactor models (10–13). Previous studies found stress as measured by the PSS to be robustly related to measures of mental health problems [e.g., depressive and anxiety symptoms (7, 14)] and indicators of positive mental health [e.g., life satisfaction (15), self-reported stressor recovery ability (16)]. Moreover, previous research showed that stress is associated with a broad range of resilience-related concepts [e.g., positive affect (17), positive reappraisal (18), self-compassion (19), self-efficacy (16), sense of coherence (20)], with higher levels of stress being associated with lower levels of resilience-related concepts. While the original scale comprised 14 items, 10-item and 4-item versions are commonly used in international research. However, there are considerable doubts about the psychometric quality of the 4-item version (21, 22), which was developed solely based on the association of single items with the long version of the PSS. The PSS-4 is interpreted by means of a total score with higher scores reflecting higher levels of general stress. However, the use of this total score has been challenged by studies examining the factorial structure of the PSS-4. For example, in a sample of English-speaking adolescents, the one-factor structure of the PSS-4 was not valid, and two distinct, albeit interrelated factors were identified (21). Similarly, another study with patients in primary healthcare services and a recent validation study on the Italian version of the PSS found the one-factor model for the PSS-4 to be inadequate (22, 23). In line, a review on different versions of the PSS concluded that the factor structure of the PSS-4 is inconclusive and its reliability is only marginally acceptable (9). Moreover, so far, for German-speaking populations two valid versions of the PSS-10 are available (7, 14), whereas the 4-item version is frequently used (24, 25) but has not yet been psychometrically validated. However, the fact that the scale is already used without being validated underlines the need for a German short version of the PSS.

The development of economic and valid short scales is of major importance for stress and resilience research. Resilience as the maintenance or regain of mental health during or after stressor exposure, inherently requires longitudinal designs (26), such as those implemented during the COVID-19 pandemic (27, 28). While longitudinal studies are essential to gain insight into resilience, these studies are highly time consuming for participants, resulting in compliance problems and high rates of dropouts (29). Thus, the development and validation of short scales may help to make resource-intense research more efficient.

At the same time, the use of short scales might result in inadequate measurement as item reduction can change the internal structure of scales, lead to lower reliability, and reduce criterion validity (30). Particularly, item reduction attempts that focus on a small number of optimization criteria—often examined in a sequential manner—are at risk to result in inadequate measurements by neglecting other psychometric properties. The use of machine learning can improve short scale development by allowing to optimize a larger number of criteria simultaneously (31). One of the most promising meta-heuristics are ant-colony-optimization (ACO) algorithms (30), which find an efficient item solution in the same way as ants find the shortest route between their nest and food source. Ants use pheromones to mark their routes, which attract other ants to the respective route. As more ants pass the shortest route per time unit pheromones accumulate faster for this route, that is, more ants are attracted to shorter routes until the majority of ants uses this route (32). ACO algorithms make use of this rationale by using virtual pheromones that increase the attractiveness of specific items (i.e., ants) associated with better psychometric properties [i.e., shorter routes (33)]. First, items are selected pseudo-randomly, and item sets are compared for their psychometric properties, with ACO increasing the pheromone levels of those items belonging to the set with the best properties. This increases the likelihood of these items being selected in the next iteration. With an increasing number of iterations, a distinct item pattern emerges resulting in an efficient short version (30).

The purpose of this study was to develop a reliable and valid German short version of the PSS. Due to previously reported psychometric shortcomings of the PSS-4 (9, 13, 21), we aimed at developing a new short version of the PSS using a state-of-the-art method for item reduction, which simultaneously optimizes several psychometric criteria. First, we briefly examined the factorial validity and the internal consistency of the PSS-4 as previously used. Second, we examined the factor structure of the PSS-14 based on the models that had been used in previous studies, that is, a one-factor model (23), a two-factor model (34), and a bifactor model (10–13). Third, we established a new short version of the scale using an ACO algorithm. Fourth, we examined the psychometric properties of this scale (i.e., reliability and measurement invariance across gender). Fifth, we used psychological network modeling to examine the construct validity of the new PSS short version. For this purpose, we explored unique associations of the PSS short version with indicators of mental health and resilience.

## 2. Materials and methods

### 2.1. Participants and procedure

The study used the WiSoPanel (35) and Clickworker for sample recruitment. The non-commercial WiSoPanel holds 14,369 German-speaking participants who live in Germany, Austria, Switzerland, or border regions of neighboring countries. The panel is not representative of the German general population but holds socioeconomically diverse respondents, who are interested in web-based studies. Respondents from the WiSoPanel received €2 as compensation for participation in the study at hand, which they could donate back to the panel. Additionally, we used the commercial crowdsourcing platform Clickworker for recruitment with the same compensation. To be eligible, respondents needed to be ≥18 years. The study was divided into two assessments to allow for the assessment of test-retest reliability. Trait measures were distributed across the two assessments to reduce respondent burden per assessment (see Supplementary material 1). Assessments took place between 19/05/2022 and 25/05/2022 (wave 1), and 23/06/2022 and 04/07/2022 (wave 2). Originally, both assessments were supposed to be open for 7 days, but due to a technical error in Clickworker, we decided to extend the assessment duration for the second assessment by 4 days. For all respondents, data were collected via the online platform SoSci Survey (36). Of the total WiSoPanel, 1,303 respondents participated in both assessments. Another 214 respondents were recruited via Clickworker, resulting in 1,517 respondents who participated in our study (see Supplementary material 2 for details). Of those, 80 were excluded due to unreasonably short answering times, which were assessed based on standard measures provided by the online platform SoSci Survey (36), with the DEG Time Index giving negative points for extremely fast completion and values ≥100 indicating low data quality (37). This resulted in a final sample of 1,437 respondents, who gave written informed consent in accordance with the Declaration of Helsinki. The study was approved by the Ethics Committee of the State Medical Association of Rhineland Palatinate, Germany (no. 2022-16402) and prospectively preregistered at PsychArchives [(38) see Supplementary material 3 for deviations from registration].

### 2.2. Instruments

#### 2.2.1. Perceived stress

We used the German version of the Perceived Stress Scale [PSS; (7)]. As only the 10-item scale was available in German and we aimed at using the full length scale for item reduction, the additional four items of the PSS-14 (5, 6) were translated to German and checked by the authors with the help of a bilingual person (see Supplementary material 4). Agreement with each item is rated on a 5-point Likert scale. The scale consists of two subscales, that is, perceived helplessness (also referred to as perceived distress) and perceived self-efficacy (also referred to as perceived coping). In the present study, the 14-item version showed very good to excellent internal consistencies, reflected in Cronbach's alpha (α) = 0.89, and McDonald's omega (ω) = 0.90.

#### 2.2.2. Mental health problems

The 4-item Patient Health Questionnaire [PHQ-4; (39)] was used to measure mental health problems. Patients rated their agreement with four statements on a 4-point Likert scale. Higher scores indicate higher mental health problems. Internal consistencies were good, α/ω = 0.88.

#### 2.2.3. Self-rated health

Self-rated health was assessed using the 1-item assessment from the German Aging Survey (40). Respondents rated their current state of health on a 5-point Likert scale, with higher scores indicating better self-rated health.

#### 2.2.4. Stressor recovery ability

The 6-item Brief Resilience Scale [BRS; (16)] was used to assess self-reported stressor recovery ability, also referred to as self-reported resilience. Respondents rated their agreement with each statement on a 5-point Likert scale. Higher scores indicate better stressor recovery ability. Internal consistencies were good, α/ω = 0.89.

#### 2.2.5. Life satisfaction

Life satisfaction was assessed using the Satisfaction with Likert Scale [SWLS; (41)]. The scale consists of five items that are rated on a 6-point Likert scale, and higher scores indicate greater life satisfaction. Internal consistencies were excellent, α/ω = 0.92.

#### 2.2.6. Affect

Affect was assessed using the Positive and Negative Affect Schedule-Trait [PANAS; (42)]. The scale contains 20 items and two subscales—positive and negative affect. All items are rated on a 5-point Likert scale. In our study, internal consistencies for both subscales were excellent; α/ω = 0.91.

#### 2.2.7. Coping

The Brief COPE (43) was used to measure the engagement in coping strategies. The 28-item measure assesses 14 coping strategies (i.e., acceptance, active coping, disengagement, denial, emotional support, humor, instrumental support, planning, positive reframing, religion, self-blame, self-distraction, substance use, and venting). Each item is rated on a 4-point Likert scale. We followed the approach by Eisenberg (44) and aggregated single coping strategies to broader categories of commonly adaptive and maladaptive strategies. Due to our focus on resilience-related factors, we used the adaptive strategies for the current analyses. Internal consistencies were good, α/ω = 0.85.

#### 2.2.8. Positive appraisal style

Positive appraisal style was assessed using the content-focused subscale of the Positive Appraisal Style Scale [PASc; (45)]. PASc assesses the typical context of one's thoughts when being challenged. The scale comprises 12 items, which are rated on a 5-point Likert scale, and higher scores indicate a more positive appraisal style. The internal consistencies of the scale were excellent, α/ω = 0.91.

#### 2.2.9. Self-efficacy

Self-efficacy was assessed used the General Self-Efficacy Short Scale [ASKU; (46)]. The 3-item scale assessed self-efficacy using a 5-point Likert scale with higher scores indicating stronger self-efficacy beliefs. The internal consistencies of the scale were excellent, α/ω = 0.91.

#### 2.2.10. Sense of coherence

Sense of coherence (SOC) was assessed using the 3-item ultra-short version of the Sense of Coherence Scale [SOC-3; (47)]. SOC-3 uses a bipolar 7-point Likert scale, and higher scores indicate a stronger SOC. The scale showed good internal consistencies, α/ω = 0.82.

#### 2.2.11. Self-compassion

Trait self-compassion was assessed using the Self-Compassion Scale [SCS-D; (48)]. The 26-item scale assesses self-compassion using a 5-point Likert scale. For our study, we used the total score, with higher scores indicating more self-compassion. The internal consistencies were excellent, α/ω = 0.93.

### 2.3. Data analyses

All analyses were performed using *R* version 4.1.2 (49).

#### 2.3.1. Missing data

For single items, missing data was replaced by mean scores per scale. In cases where more than one item was missing per scale, missing items were removed per scale.

#### 2.3.2. Descriptive analysis

To examine normality at single-item level for the PSS, we used Shapiro-Wilk tests, skewness, and kurtosis. Non-normality was indicated by a significant Shapiro-Wilk test and skewness or kurtosis exceeding the range from −1.5 to 1.5 (50).

#### 2.3.3. Factor analysis

First, we used confirmatory factor analysis (CFA) to examine the factorial validity of the German version of the PSS-4 as used in previous studies [(24, 25); i.e., a one-factor model]. Second, as this model provided evidence for insufficient model fit, we proceeded with the development of our new short version starting with the German version of the PSS-14. CFA were used to compare a one-factor model, a two-factor model and a bifactor model for the PSS-14 using the *R* package *lavaan* (51). For estimation, the Weighted Least Squares Mean and Variance (WLSMV) adjusted was used to account for ordinal data at single-item level, while the Comparative Fit Index (CFI) and Root Mean Square Error of Approximation (RMSEA) were used for model evaluation [acceptable fit: CFI ≥ 0.90, RMSEA ≤ 0.08; good fit: CFI ≥ 0.93, RMSEA ≤ 0.06; excellent fit: CFI ≥ 0.96, RMSEA ≤ 0.05; (52)]. The one- and two-factor model as well as the bifactor model were compared with χ^2^ difference testing as direct comparisons of model fit indices are not advisable for WLSMV. For sensitivity analyses, we re-estimated our bifactor model using (robust) maximum likelihood estimations, which had been used in previous studies on German versions of the PSS (7, 10, 14).

Additionally, we examined the gender-specific measurement invariance of the PSS. Measurement invariance is a crucial prerequisite for group comparisons but only for some versions of the PSS measurement invariance has been established (7, 53, 54). Thus, we used multiple group confirmatory factor analysis [MGCFA; (55)] as a straightforward procedure of sequentially constraining measurement parameters to be equal across groups with increasing levels of invariance (i.e., factor loadings, thresholds, and residual variances). The different levels of invariance were assessed by subsequently comparing measurement models from least to most restrictive: First, *configural* invariance is examined by restricting the factor structure to equality between groups but freely estimating the model parameters. Evidence of configural invariance shows that the same subset of items is associated with the same constructs across groups. However, the values of the parameters may vary, and separate analyses for the groups are recommended. In a next step, *metric* invariance requires the factor loadings to be constrained to equality across groups. The item thresholds and residual variance are still estimated freely at this level. If metric invariance can be established, it implies that the strength of the relationship between the items and the latent construct it the same across groups. Latent variances and covariances can be compared at this level. For *scalar* invariance item thresholds are restricted to be equal (in addition to the factor loadings), while the residuals are still estimated freely. This level of invariance implies that the range of responses given to each item is the same across groups. This allows for between-group comparisons of latent factor means. Finally, *strict* invariance requires the additional fixation of item residuals to equality across groups, which would mean that the observed differences are only accounted for by true between-group differences. Thus, unbiased observed and latent comparisons can be made for variances, covariances, and means. Again, due to the use of the WLSMV estimator, we compared the models with χ^2^ difference testing, with a significant χ^2^ difference test indicating the more constrained model resulted in a significant decrease of model fit (i.e., the respective level of measurement invariance could not be achieved).

#### 2.3.4. Item reduction

Items for the short scale were selected using a ACO algorithm (30, 56). The algorithm requires a priori definition of the scale length and the criteria that should be used for item selection. For the evaluation of the model fit, we included the CFI and RMSEA. The optimization criteria were met if the CFI was ≥0.96 and RMSEA was ≤ 0.05. They were integrated equally weighted to estimate the pheromone of the model fit. Additionally, ω was introduced as a reliability coefficient for estimating pheromone levels. The optimization criterion was set at ω ≥ 0.85. Furthermore, test-retest reliability was added as an optimization criterion using the *R* package *irr* (57) and an intra-class correlation (ICC) ≥ 0.70 as a cut-off indicating good test-retest reliability. Finally, we included correlations with covariates to account for construct validity by ensuring that the selected items are anchored in an established theoretical framework. Building on previous work, we chose correlations of *r* = 0.59 with depressive symptoms, *r* = −0.53 with stressor recovery ability, and *r* = 0.44 with self-efficacy each with a tolerance of ±0.15 as target criteria (14, 16). The optimization criterion for associations with covariates was established as the mean of the differences of each correlation with the respective target criteria.

Once the initial parameters (i.e., number of items and ants, evaporation rate, optimization criteria, estimation of pheromone level and update) are fixed, the algorithm runs through several iterations until the convergence criterion is met. For the first run, the pheromone level is equal across items, resulting in the pseudo-random item selection. The selected item sets are evaluated based on the optimization criteria and the pheromone level (ϕ) is estimated for all models. The overall pheromone level was defined as a sum of pheromone values from the individual optimization criteria. The hitherto best solution can then be determined (iteration 1) or the best pheromone level of the current item selections can be compared to the hitherto best pheromone from previous iterations. If the current item set has a higher pheromone value, the hitherto best pheromone is updated. The pheromone weight is added to the initial pheromone. Then, this procedure is repeated until the convergence criterion is met.

#### 2.3.5. Partial correlational networks

Network analyses were performed using the *R* packages *bootnet* (58), *qgraph* (59), and *huge* (60). We calculated cross-sectional partial correlation network models using a high-dimensional undirected graph estimation (huge) with mental health outcomes, resilience-related concepts, and the newly derived PSS factors as variables (i.e., nodes). Interrelations between nodes (i.e., edges) represent partial correlations. The estimation uses the Least Absolute Shrinkage and Selection Operator [LASSO; (61)] method to shrink small (i.e., less relevant) edge weights to zero. To choose the final network model, we used Extended Bayesian Information Criterion (EBIC, hyperparameter = 0.25) and applied bootstrapping with 1,000 draws to examine the robustness of edge weights based on 95% confidence intervals (CIs). We used correlation stability coefficients to examine centrality stability, and strength, closeness and betweenness as centrality indices describing the role of each node in the network.

## 3. Results

### 3.1. Analysis sample

The final sample comprised 1,437 respondents with an average age of 54.27 years (*SD* = 14.54) and 52.8% being female (see Table 1). The overall number of missing data was very low (≤0.1% per scale).

**Table 1:** Sample characteristics.

	**Total sample (*n* = 1,437)**
Age [*M* (*SD*), range]	54.27 (14.54), 19–88 years
**Gender (** ***n*** **, %)**	
Women	759 (52.8%)
Men	675 (47.0%)
Non-binary	2 (0.1%)
Not reported	1 (0.1%)
**Educational level (** ***n*** **, %)**	
No school degree	5 (0.3%)
Nine years of school	149 (10.4%)
Ten years of school	436 (30.3%)
A-level exam	312 (21.7%)
University degree	490 (34.1%)
Doctoral degree	45 (3.1%)
**Country (** ***n*** **, %)**	
Germany	1,371 (95.4%)
Austria	41 (2.9%)
Switzerland	17 (1.2%)
Other	8 (0.6%)
**Recruitment (** ***n*** **, %)**	
WiSoPanel	1,238 (86.2%)
Clickworker	199 (13.8%)

### 3.2. Normality of the PSS-14

Shapiro-Wilk tests were significant for all PSS-14 items, all *p*s < 0.001. However, this may also reflect high statistical power due to our overall large sample making the Shapiro-Wilks test highly sensitive even for small and less relevant deviations from normality. Thus, we inspected skewness and kurtosis, with skewness ranging from −0.06 to 0.77, and kurtosis ranging between −0.82 and 0.62, indicating no strong deviations from normality.

### 3.3. One-factor model of the former PSS-4

Studies employing the German version of PSS-4 used a total score for interpretation, which implies a one-factor structure of the scale. Thus, we examined the fit of a one-factor model using the items of the PSS-4, finding an inacceptable model fit, χ^2^(6) = 1,164.73; CFI = 0.85; RMSEA = 0.244, while the internal consistencies of the PSS-4 were acceptable, ω/α = 0.76. These findings further supported our project aim of developing a new short version of the PSS simultaneously optimizing multiple psychometric criteria.

### 3.4. Factor analysis and measurement invariance of the PSS-14

The CFA for a one-factor model of the 14-item PSS did not show good fit, χ^2^(77) = 4,707.0; CFI = 0.817; RMSEA = 0.205, and the two-factor model could barely be considered acceptable, χ^2^(76) = 2,308.6; CFI = 0.912; RMSEA = 0.143. The χ^2^-difference testing confirmed the advantage of the two-factor model, χ^2^(2) = 777.1; *p* < 0.001. We then employed a bifactor model with one general factor and two specific factors (i.e., summarizing positively and negatively worded items^1^), which showed a more acceptable fit; χ^2^(65) = 856.70; CFI = 0.96; RMSEA = 0.094.^2^ Again, the χ^2^-difference testing confirmed the advantage of the bifactor model over the two-factor model, χ^2^(13) = 978.08; *p* < 0.001. Thus, we used the bifactor model for subsequent analyses.

The results of the measurement invariance analysis based on the bifactor model are in Table 2. We were unable to establish metric invariance with a fully restricted model. However, modification indices suggested freeing the factor loadings of items 4, 5, 9, and 11 for the general factor, resulting in partial metric invariance for the PSS-14. Further analyses did not support full or partial scalar invariance.

**Table 2:** Invariance testing between gender for the PSS-14 based on the bifactor model.

**Model**	**χ^2^(df)**	**CFI**	**RMSEA**	**Model comparison**	**χ^2^(Δdf)**	***p***
1) Configural	978.9 (126)	0.966	0.097			
2a) Metric	909.8 (179)	0.976	0.076	1 vs. 2a	140.1 (53)	<0.001
2b) Metric (partial)	842.9 (172)	0.973	0.074	1 vs. 2b	62.4 (46)	0.054
3) Scalar (partial)	1,045.5 (186)	0.965	0.080	2b vs. 3	124.9 (14)	<0.001

CFI, Comparative Fit Index; df, degrees of freedom; RMSEA, Root Mean Square Error of Approximation.

### 3.5. Item reduction

For the short scale, we compared a 4-item and a 6-item version. Both versions were restricted to equal number of items per factor (i.e., a PSS-2&2 and a PSS-3&3 version). The ACO algorithm completed five runs per version, each with 40 ants, an evaporation rate of 0.70 and 30 iterations. The individual optimization criteria (model fit, ω, correlations with covariates, ICCs) contributed equally to the overall pheromone value, thus, the best solution was selected as the solution with the highest overall pheromone value based on five runs. The runs for both versions were highly consistent, with the algorithm reproducing the best solution four out of five times. In line with our selection criteria, both versions showed excellent fit for a two-factor model, high internal consistency, moderate to good test-retest reliability, and expected correlations with covariates (see Table 3).

**Table 3:** Measurement models for the PSS-2&2 and PSS-3&3.

**Model**	**CFI**	**RMSEA**	**ω**	***ICC***	**Mental health problems**	**Stressor recovery ability**	**Self-efficacy**	**Mean factor loadings**
PSS-2&2	0.998	0.054			0.69	−0.61	−0.54	0.81
Helplessness			0.85	0.74				
Self-efficacy			0.84	0.64				
PSS-3&3	0.996	0.043			0.69	−0.62	−0.53	0.76
Helplessness			0.85	0.76				
Self-efficacy			0.82	0.55				

CFI, Comparative Fit Index; ICC, intra-class correlation; RMSEA, Root Mean Square Error of Approximation. Correlations with covariates were estimated across all items as previous versions did not assume a two-factor structure.

The items of each short scale and their respective factor loadings are in Table 4. As both short scales met our previous defined criteria in a comparable manner, we opted for the shorter and thus more economic PSS-2&2. The scale achieved full metric invariance between gender and by freeing the intercept of item 6, partial scalar invariance (see Table 5).

**Table 4:** Item selection for the PSS-14, PSS-2&2, and PSS-3&3.

**Items**	**PSS-14**		**PSS-2&2**		**PSS-3&3**	
	**Self-efficacy**	**Helplessness**	**Self-efficacy**	**Helplessness**	**Self-efficacy**	**Helplessness**
1		0.78		0.76		0.83
2		0.87				
3		0.82				0.88
4	0.65				0.70	
5	0.75				0.84	
6	0.88		0.85			
7	0.79		0.85			
8		0.74				
9	0.56				0.59	
10	0.90					
11		0.69		0.77		0.72
12		0.50				
13	0.54					
14		0.87				

Original items and German translations can be found in Supplementary material 4.

**Table 5:** Invariance testing between gender for the PSS-2&2.

**Model**	**χ^2^(df)**	**CFI**	**RMSEA**	**Model comparison**	**χ^2^(Δdf)**	***p***
1) Configural	4.5 (2)	0.999	0.042			
2) Metric	13.7 (12)	1.0	0.014	1 vs. 2	10.4 (10)	0.407
3a) Scalar	27.0 (14)	0.997	0.036	2 vs. 3a	8.4 (2)	0.015
3b) Scalar (partial)	20.5 (13)	0.998	0.028	2 vs. 3b	3.9 (1)	0.047
4) Strict (partial)	70.5 (17)	0.986	0.066	3b vs. 4	38.6 (4)	<0.001

CFI, Comparative Fit Index; df, degrees of freedom; RMSEA, Root Mean Square Error of Approximation.

When we checked whether a bifactor model would also show superior fit for the short version of the PSS, models did not converge. The same applied to a two-factor model with a second-order general factor.

### 3.6. Network models

#### 3.6.1. Bivariate correlations

**Figure 1:**
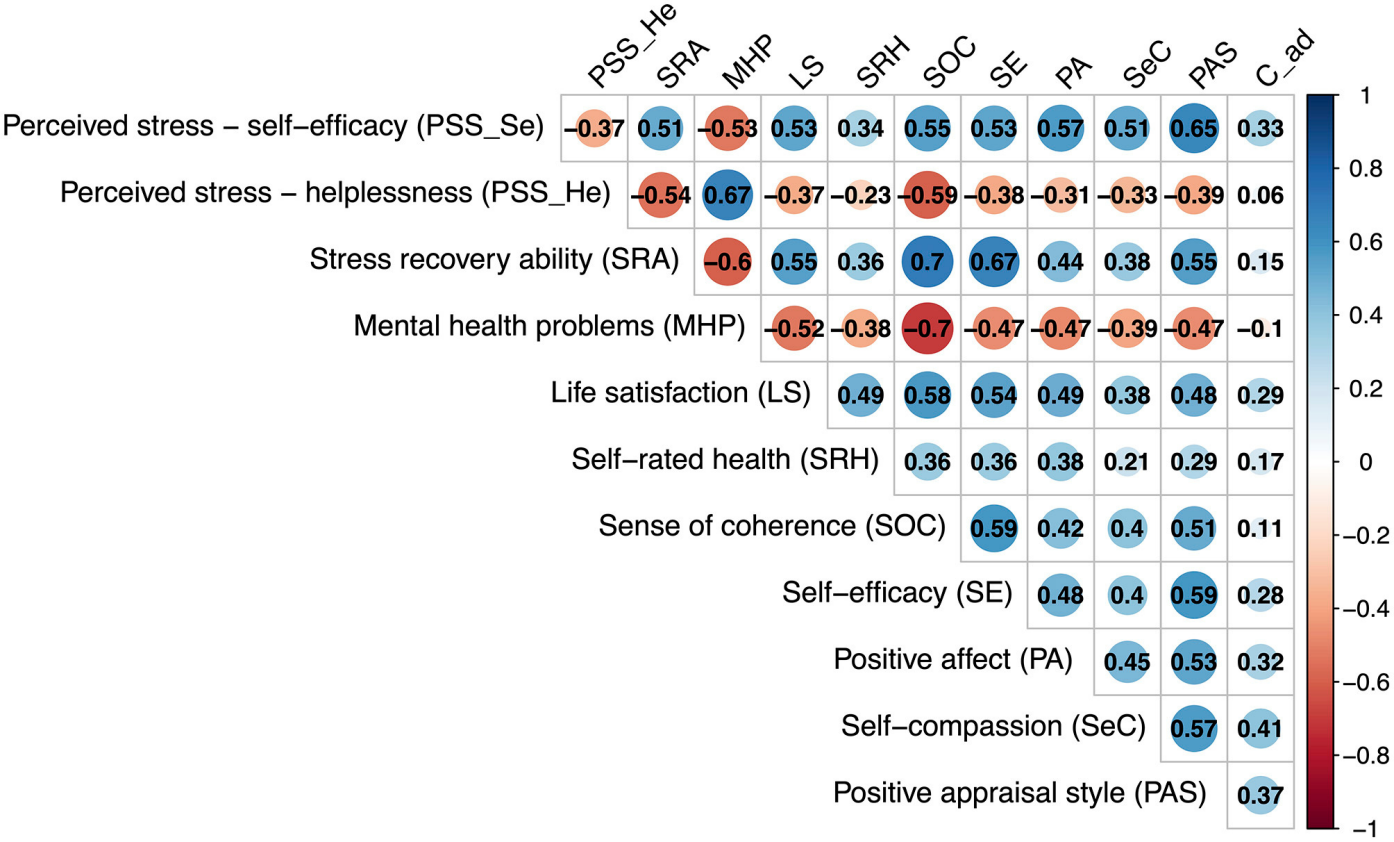
Pearson correlations. All correlations were significant at *p* < 0.05. C_ad, adaptive coping.

Figure 1 shows bivariate correlations between study variables. Both factors of the PSS-2&2 factors were negatively interrelated, *r* = −0.37, *p* < 0.001.

#### 3.6.2. Network models

**Figure 2:**
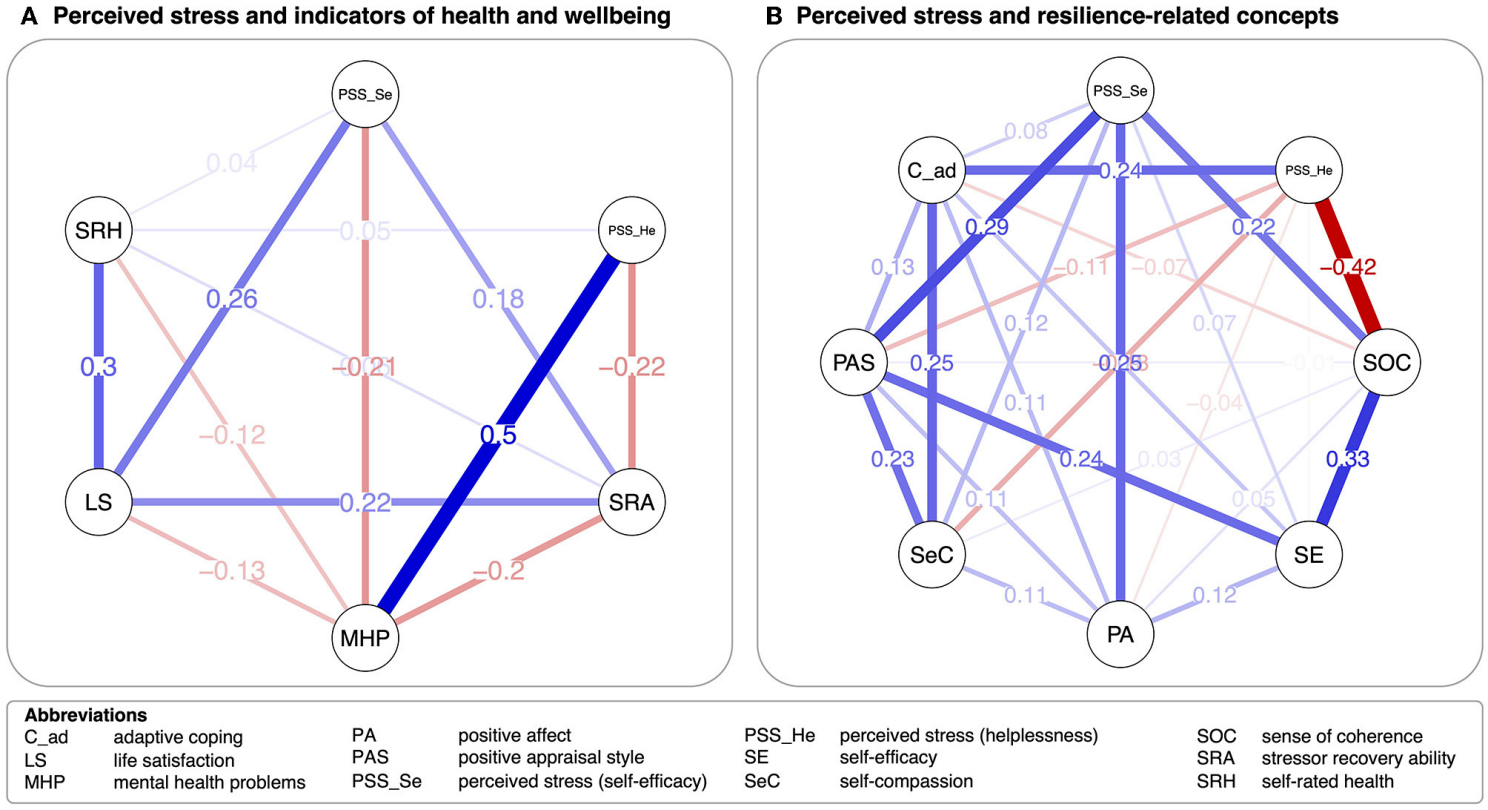
Network models on indicators of **(A)** mental health and **(B)** resilience-related concepts. Lines (i.e., edges) reflect partial correlations. Blue lines indicate positive relationships, red lines negative relationships. Wider lines represent stronger associations.

Figure 2 displays network models comprising the PSS factors together with indicators of mental health (see Figure 2A) and resilience-related concepts (see Figure 2B). In the former model, 13 of 15 possible edges were included. Overall, the PSS-2&2 self-efficacy factor showed weaker links than the helplessness factor. The PSS-2&2 self-efficacy factor showed positive partial correlations with stressor recovery ability, *r* = 0.18, life satisfaction, *r* = 0.26, and self-rated health, *r* = 0.04, which was also found for the PSS-2&2 helplessness factor, *r* = 0.05. Besides this link, the PSS-2&2 helplessness factor showed unique positive association with mental health problems, *r* = 0.50, and a negative relationship with stressor recovery ability, *r* = −0.22. Centrality indices and bootstrapped CIs of edge weights are in Supplementary material 5.

In the network model including resilience-related factors, 26 of 28 links survived LASSO regularization. Again, associations for the PSS-2&2 self-efficacy factor were weaker than for the helplessness factor. The PSS-2&2 self-efficacy factor showed the strongest unique positive links to positive affect, *r* = 0.25, positive appraisal style, *r* = 0.29, sense of coherence, *r* = 0.22, and self-compassion, *r* = 0.12. The PSS-2&2 helplessness factor shared negative links with SOC, *r* = −0.42, self-compassion, *r* = −0.13, and positive appraisal style, *r* = −0.11. The strongest positive link emerged with adaptive coping strategies, *r* = 0.24. Centrality indices and bootstrapped CIs of edge weights are in Supplementary material 5.

## 4. Discussion

In the current study, we developed a reliable and valid German short version of the PSS using an ACO algorithm for item reduction. Based on a bifactor structure of the PSS-14 and previous findings that suggested that a one-factor structure for short versions of the PSS was not adequate, we aimed at establishing a two-factor short version of the PSS, the new PSS-2&2. Both subscales demonstrated good to excellent internal consistencies and test-retest reliabilities over 4 weeks. Using psychological network modeling, we derived information on construct validity by visualizing associations of the PSS-2&2 factors with indicators of mental health problems and resilience. We found higher levels of the helplessness factor to be associated with more mental health problems as well as lower stressor recovery ability and SOC. Higher levels of the self-efficacy factor were associated with lower mental health problems as well as more stressor recovery ability, life satisfaction, positive appraisal, positive affect, self-compassion, and SOC.

Interestingly, in our sample, neither the PSS-14 nor the PSS-10 demonstrated good factorial fit and were at the most close-to-acceptable, with the bifactor model including a general factor and two specific factors (for positively and negatively worded items) showing the best fit. While our sample showed similar characteristics compared to other validation samples used for German versions of the PSS, our modeling decisions were slightly different: While Klein et al. (14) and Schneider et al. (7) used maximum likelihood estimators and Reis et al. (10) applied robust maximum likelihood estimators, we used WLSMV to account for ordinal data at single-item level. We deemed this approach more appropriate for PSS items, however, when we applied robust maximum likelihood estimators for sensitivity analyses, model fit of our bifactor model increased from close-to-acceptable to the good to excellent range. Thus, the reason for the worse fit of the long version of the PSS may lie in the use of a more conservative estimator in our study.

For the PSS short version, our item reduction process provided evidence for a two-factor structure. In case we did not force the algorithm to select items from two factors, the ACO solution selected only items from the helplessness factor (see Supplementary material 6). These findings tie in with previous studies questioning the one-factor structure of PSS short versions (9, 21, 22) and further challenge the use of the PSS short version as a unidimensional measure of stress. This was further evidenced by our network models that showed heterogeneous associations for both factors, with the helplessness factor having the strongest positive link with mental health problems and a substantial negative association with SOC, a resilience factor, which showed substantial links to different health indicators (62–64). By contrast, the self-efficacy factor showed more differential associations and was stronger related to positive mental health indicators like life satisfaction and stressor-recovery ability. With respect to resilience-related concepts, the self-efficacy factor showed strong associations with positive appraisal style, positive affect, and SOC, but interestingly, not with self-efficacy in our sample. These associations also challenge the naming of the factors, whereby perceived distress and coping might be more appropriate than the commonly used names (65).

The two-factorial structure of the PSS-2&2 is of major importance for researchers interested in using the PSS in their studies. Based on our results, researchers should not use total scores of the PSS-2&2 (or the previously used unidimensional PSS-4) as indicators of overall perceived stress but examine the factors helplessness (or perceived distress) and self-efficacy (or coping) separately. For the PSS-14, we found a bifactor structure, which allows for both the use of total and subscale scores. In line with previous recommendations (10), researchers should make informed decisions on whether they want to use the PSS-2&2 or long versions of the PSS (i.e., the PSS-10 and PSS-14). In cases where researchers are interested in overall perceived stress, one may rather use the total score of the PSS long versions. However, researchers should keep in mind that this total score comprises both the general factor along with unmodeled variance accounted for by the specific factors. In cases where researchers are interested in the PSS factors helplessness (or perceived distress) and self-efficacy (or coping), researchers may use the PSS-2&2.

A shortcoming of the long version of the PSS was the missing measurement invariance between gender in our sample. This finding contrasts with previous studies (53, 54), but may point to a shortcoming of the German long version for which measurement invariance between gender has only been examined in a single study using a bifactor model (10), but not in other validation studies (7, 14). In our sample, we were not able to establish measurement invariance for the bifactor model of the PSS-14 beyond configural invariance. These results suggest that gender-specific group comparisons with the PSS-14 may be biased and should be interpreted with caution. Preferably, separate gender-specific analyses should be conducted when using this scale.

By contrast, we found full metric measurement invariance across gender for the PSS-2&2 and at least partial scalar invariance for the PSS-2&2. The minimal adjustments needed for the PSS-2&2 suggest that the lack of full scalar invariance for the scale was an artifact of the specific sample. Therefore, the PSS-2&2 might be a reliable scale for between-gender stress comparisons. However, future studies need to replicate our findings on measurement invariance of the PSS-2&2. We did not aim at examining measurement invariance over time for the PSS-2&2 in the current study, but future studies should address this together with other aspects of measurement invariance (e.g., between age groups).

### 4.1. Strength and limitations

The current study was the first to develop a valid short version of the German PSS using a large and heterogeneous sample. In contrast to previous short versions of the PSS (6), we used an ACO algorithm for item reduction, which ensured that our short version was simultaneous optimized for multiple criteria including reliability and construct validity. Thereby, our study is another use case for ACO-based item reduction for health measures. Moreover, we used psychological network modeling that provided further insights into unique associations of the PSS factors with other constructs.

However, our study also comes with limitations. While the ACO algorithm allowed for optimizing several criteria simultaneously, it may not necessary result in the best solution (30). We addressed this problem by running the item selection procedure five times, with four out of five runs resulting in the same item set. However, we cannot exclude that other solutions may have resulted in superior properties or that other item combinations may have led to comparably good psychometric properties. However, by contrast to previous short versions of the PSS, we employed a more sophisticated approach to item reduction by simultaneously optimizing multiple criteria. In line with previous research (9, 22, 23), we found *post-hoc* evidence that using the PSS-4 as one-factor measure is inadequate, however, we explicitly did not aim at providing a full validation of the PSS-4 as used in previous research due to the previously identified problems with the PSS-4. Moreover, our sample was not representative of the German general population. The WiSoPanel (35) holds socio-economically diverse persons, however, these tend to be older, more likely to be female and better educated than the general population (66, 67). Such differences may also apply to respondents recruited via Clickworker. Thus, our findings need to be replicated in a representative sample as well as in specific (non-)clinical populations. Such studies may also allow to derive population norms. Moreover, we did not use items for attention checking in this study. We aimed to handle this problem by reducing the duration of assessments, which was ~10–12 min per assessment wave and by excluding respondents with unreasonably short answering times, which may reflect low levels of attention. However, we cannot exclude that some respondents were inattentive when completing the measures used in this study.

## 5. Conclusion

The PSS is among the most used psychometric scales in stress and resilience research. However, yet a reliable and valid German short version of the scale was missing. The present study aimed at addressing this gap and provided a 4-item short version with a 2-factor structure, the PSS-2&2. Future resilience research may use this scale to assess self-perceived stress and may derive further knowledge on the adequate naming of its factors. Moreover, our study may also inspire future international validation studies of short versions of the PSS, which may benefit from using machine learning to simultaneously optimize a broad range of psychometric criteria.

## Data availability statement

The raw data supporting the conclusions of this article will be made available by the authors, without undue reservation.

## Ethics statement

The studies involving human participants were reviewed and approved by Ethics Committee of the State Medical Association of Rhineland Palatinate, Germany (no. 2022-16402). The patients/participants provided their written informed consent to participate in this study.

## Author contributions

AG, AM (lead), LB, KL, MW, OT, SB, and SS (lead) contributed to conception and design of the study. AG, AM, and LB (lead) organized the database. AM (lead) and SS performed the statistical analysis. SS (lead) and AM wrote the first draft of the manuscript. All authors contributed to manuscript revision, read, and approved the submitted version.

## Footnotes

^1^Reis et al. (10) found one general factor and two specific factors for the German version of the PSS-10, with two items not being included in these specific factors. As we used the 14-item version for our analyses, we tested a bifactor model, in which we assigned all items to the positively and negatively worded specific factors as this has been done in previous research.

^2^When we used robust maximum likelihood estimations (MLR): χ^2^(65) = 373.94; CFI = 0.96; RMSEA = 0.059.
