# Supplementary material for: The Perceived Stress Scale 2&2: a two-factorial German short version of the Perceived Stress Scale
Source: Front Psychiatry. 2023 Jul 6;14:1195986. doi: 10.3389/fpsyt.2023.1195986 (PMC10358735; doi:10.3389/fpsyt.2023.1195986)
Supplement: Supplementary file 1 [file Data_Sheet_1.docx]

Supplementary Material

The Perceived Stress Scale 2&2: A Two-Factorial German Short Version of the Perceived Stress Scale

**Sarah K. Schäfer, Lisa von Boros, Anja S. Göritz, Sophie Baumann, Michèle Wessa,
Oliver Tüscher, Klaus Lieb, & Anne Möhring**

*** Correspondence:**Sarah K. Schäfer
sarah.schaefer@lir-mainz.de

**Table of Contents**

[SM1. Time plan of assessments 2](#_Toc138417870)

[SM2. Study flow chart 3](#_Toc138417871)

[SM3. Deviations from the preregistration (https://doi.org/10.23668/psycharchives.6665) 4](#_Toc138417872)

[SM4. Perceived Stress Scale 14 (English version and German translation) 6](#_Toc138417873)

[SM5. Details on network analyses 8](#_Toc138417874)

[SM6. Additional results on item reduction without two-factor structure 9](#_Toc138417875)

[SM7. The PSS-2&2 (German version) 10](#_Toc138417876)

# SM1. Time plan of assessments

| **Table 1.** Time plan of assessments |  | Timepoint | |
| --- | --- | --- | --- |
| Questionnaire | # Items | 1 | 2 |
| Trait Self-Compassion Scale (Hupfeld & Ruffieux, 2011) | 26 | x | x |
| Positive and Negative Affect Schedule (PANAS; Breyer & Bluemke, 2016) | 20 | x |  |
| Brief COPE (Knoll, Rieckmann & Schwarzer, 2005) | 28 | x |  |
| Satisfaction with Life Scale (SWLS; Janke & Glöckner-Rist, 2012) | 5 |  | x |
| Brief Resilience Scale (Chmitorz et al., 2018) | 6 |  | x |
| General Self-efficacy short scale (ASKU; Beierlein et al., 2014) | 3 |  | x |
| Sense of Coherence Scale ultrashort form (SOC-3; Schmalbach et al., 2020) | 3 |  | x |
| State optimism measure (SOM; Millstein et al., 2019) | 7 | x |  |
| Subjective Health Item from Deutscher Alterssurvey 2017 (DEAS; Engstler et al., 2017) | 1 |  | x |
| State Anxiety Scale of the STAI (Laux et al., 1981) | 20 | x |  |
| Patient Health Questionnaire (PHQ-4; Löwe et al., 2010) | 4 |  | x |
| Perceived Stress Scale (14-item version by Cohen et al., 1983) | 14 | x |  |

# SM2. Study flow chart

**Figure 1.** Information on sample selection

*Note*. Unreasonably short completion time was defined according to the Deg Time Index provided by SoSci Survey, with the Deg Time Index giving negative points for extremely fast completion and values ≥ 100 indicating low data quality. Respondents with Deg Time Indices ≥ 100 were excluded from our analyses.

# SM3. Deviations from the preregistration (https://doi.org/10.23668/psycharchives.6665)

**Table 2**. Differences between preregistration and final manuscript

|  | Preregistration | Final manuscript |
| --- | --- | --- |
| 1 | (In “M14 Study Procedures”) …the second part of the study will be online for one week, and participants can fill the questionnaire out whenever they want within that period. | (In “Methods: Participants and Procedure”) Originally, both assessments were supposed to be open for 7 days, but due to a technical error in Clickworker, we decided to extend the assessment duration for the second assessment by 4 days. |
| 2 | (In „Measured Variables – with matching hypotheses, if applicable“)  - Emotional approach coping strategies: emotional support, acceptance, positive  reframing, humour (H2d)  - Problem-focused coping strategies: active coping, planning, instrumental support, religious coping (H2e)  - Maladaptive coping: behavioral disengagement, denial, self-blame, substance use (H3c) | (In “Measurements – Indicators of Mental Health”) For the current study, we followed the approach by Eisenberg (2012) and aggregated single coping strategies to broader categories of commonly adaptive strategies.  *Line of reasoning*: In our preregistration, we planned to use the above-mentioned scales for the Brief COPE because we found evidence in the literature for these scales to be associated with self-compassion (see Ewert et al., 2021). However, we realized that the usage of these scales was not the most common way for the aggregation of scales for the Brief COPE (Solberg et al., 2022), so we adjusted it accordingly for the PSS version and our network models on construct validity. |
| 3 | (In “AP6 Statistical models”) Multiple optimization criteria will be included simultaneously to determine the optimal solution for the short scales, focusing on correlations with related constructs (…), fit indices (Comparative Fit Index: …); Root Mean Square Error of Approximation: (…), and reliability (Mc Donald’s omega: (…)). | (In “Data analyses – Item reduction”) For the evaluation of the model fit, we included the CFI and RMSEA. (…) Additionally, ω was introduced as a reliability coefficient for estimating pheromone levels. (…) Furthermore, test-retest reliability was added as an optimization criterion using the *R* package *irr* (Gamer et al., 2019) and an intra-class correlation (ICC) ≥. 70 as a cut-off indicating good test-retest reliability. Finally, we included correlations with relevant covariates to account for convergent construct validity by ensuring that the selected items are anchored in an established theoretical framework.  *Line of reasoning*: As part of the discussion process within the project team, we agreed on including temporal stability as additional optimization criterion. This has been done as we assessed perceived stress within a short period of time without consistent stressor exposure. As the PSS scale is not designed to be a state measure, we deemed a substantial test-retest reliability as appropriate criterion. |
| 4 | (In “AP6 Statistical models”) The factor structure of the short forms of (…) PSS (...) will be tested with single-factor CFAs. | (In “Results - Factor Analysis and Measurement Invariance”) The χ^2^-difference testing confirmed the advantage of the two-factor model, χ^2^(2) = 777.1; *p* < .001. Thus, we used the two-factor model for subsequent analyses.  *Line of reasoning*: As we found a 2-factor version to show superior fit, all analyses used 2-factor models. |
| 5 | (In “AP8 Exploratory analysis“)  A network model will be calculated with all psychological variables included. | *Line of reasoning*: We only included those variables in the network models that were related to perceived stress in the previous literature. Therefore, we did not include state-measures, i.e. state self-compassion, state optimism, and state anxiety. Also, self criticism was not included in our network-model as this was solely assessed due to its association with self-compassion. |

# SM4. Perceived Stress Scale 14 (English version and German translation)

| **# Item** | **English version** | **German translation** |
| --- | --- | --- |
| Likert scale | 0 = never; 1 = almost never, 2 = sometimes; 3 = fairly often;  4 = very often | 0 = nie; 1 = fast nie; 2 = manchmal;  3 = ziemlich oft; 4 = sehr oft |
| 1 | In the last month, how often have you been upset because of something that happened unexpectedly? | Wie oft waren Sie im letzten Monat aufgewühlt, weil etwas unerwartet passiert ist? |
| 2 | In the last month, how often have you felt that you were unable to control the important things in your life? | Wie oft hatten Sie im letzten Monat das Gefühl, nicht in der Lage zu sein, die wichtigen Dinge in Ihrem Leben kontrollieren zu können? |
| 3 | In the last month, how often have you felt nervous and “stressed”? | Wie oft haben Sie sich im letzten Monat nervös und gestresst gefühlt? |
| 4* | In the last month, how often have you dealt successfully with day to day problems and annoyances? | Wie oft haben Sie im letzten Monat Alltagsstress erfolgreich bewältigt? |
| 5* | In the last month, how often have you felt that you were effectively coping with important changes that were occurring in your life? | Wie oft hatten Sie im letzten Monat das Gefühl, wichtige Veränderungen in Ihrem Leben erfolgreich zu bewältigen? |
| 6 | In the last month, how often have you felt confident about your ability to handle your personal problems? | Wie oft waren Sie im letzten Monat zuversichtlich, dass Sie fähig sind, Ihre persönlichen Probleme zu bewältigen? |
| 7 | In the last month, how often have you felt that things were going your way? | Wie oft hatten Sie im letzten Monat das Gefühl, dass sich die Dinge zu Ihren Gunsten entwickeln? |
| 8 | In the last month, how often have you found that you could not cope with all the things that you had to do? | Wie oft hatten Sie im letzten Monat den Eindruck, nicht all Ihren anstehenden Aufgaben gewachsen zu sein? |
| 9 | In the last month, how often have you been able to control irritations in your life? | Wie oft waren Sie im letzten Monat in der Lage, ärgerliche Situationen in Ihrem Leben zu beeinflussen? |
| 10 | In the last month, how often have you felt that you were on top of things? | Wie oft hatten Sie im letzten Monat das Gefühl, alles im Griff zu haben? |
| 11 | In the last month, how often have you been angered because of things that were outside of your control? | Wie oft haben Sie sich im letzten Monat über Dinge geärgert, über die Sie keine Kontrolle hatten? |
| 12* | In the last month, how often have you found yourself thinking about things that you have to accomplish? | Wie oft haben Sie im letzten Monat darüber nachgedacht, was Sie alles erreichen müssen? |
| 13* | In the last month, how often have you been able to control the way you spend your time? | Wie oft hatten Sie im letzten Monat die Kontrolle darüber, wie Sie Ihre Zeit verbringen? |
| 14 | In the last month, how often have you felt difficulties were piling up so high that you could not overcome them? | Wie oft hatten Sie im letzten Monat das Gefühl, dass sich so viele Schwierigkeiten angehäuft haben, dass Sie diese nicht überwinden konnten? |

* Items only included in the Perceived Stress Scale 14 (PSS-14).

Based on

Schneider, E. E., Schönfelder, S., Domke-Wolf, M., & Wessa, M. (2020). Measuring stress in clinical and nonclinical subjects using a German adaptation of the Perceived Stress Scale. *International Journal of Clinical and Health Psychology*, *20*(2), 173–181. https://doi.org/10.1016/j.ijchp.2020.03.004

# SM5. Details on network analyses

**Figure 2**. Centrality indices for the network models of perceived stress, indicators of health and wellbeing as well as resilience-related concepts

*Note*. Centrality indices for both network models (z standardized). Strength reflects the sum of the edge weights connected to the respective node. Closeness describes the number of steps that is required to access every other node from a given node. Betweenness is defined as the number of shortest paths that go through a given node to connect other nodes.

**Figure 3**. Correlation stability and bootstrapped confidence intervals of edge weights for the network models of perceived stress, indicators of health and wellbeing as well as resilience-related concepts

*Note*. Stability was sufficient for both network models for strength and closeness. Stability for betweenness was lower, but still sufficient. Narrow 95% bootstrapped confidence intervals of edge weights indicate sufficient stability allowing for the interpretation of edge weights.

# SM6. Additional results on item reduction without two-factor structure

**Table 3**. Item selection for the PSS-14, PSS-4 and PSS-6 (without restrictions on PSS factors)

| Items | PSS-14 | | PSS-4 | | PSS-6 | |
| --- | --- | --- | --- | --- | --- | --- |
|  | Self-efficacy | Helplessness | Self-efficacy | Helplessness | Self-efficacy | Helplessness |
| 1 |  | .78 |  | .73 |  | .85 |
| 2 |  | .87 |  | .83 |  | .86 |
| 3 |  | .82 |  | .58 |  | .71 |
| 4 | .65 |  |  |  |  |  |
| 5 | .75 |  |  |  |  |  |
| 6 | .88 |  |  |  |  |  |
| 7 | .79 |  |  |  |  |  |
| 8 |  | .74 |  |  |  |  |
| 9 | .56 |  |  |  |  |  |
| 10 | .90 |  |  |  |  |  |
| 11 |  | .69 |  | .88 |  | .57 |
| 12 |  | .50 |  |  |  | .80 |
| 13 | .54 |  |  |  |  |  |
| 14 |  | .87 |  |  |  | .83 |

*Note*. Original items and German translations can be found in SM4.

**Table 4**. Measurement models for the PSS-4 and PSS-6

| Model | CFI | RMSEA | ω | ICC | Mental health problems | Stressor recovery ability | Self-efficacy | Mean factor loadings |
| --- | --- | --- | --- | --- | --- | --- | --- | --- |
| PSS-4 | 1.0 | 0 | .89 | .55 | .72 | -.58 | -.43 | .82 |
| PSS-6 | .999 | .044 | .90 | .48 | .74 | -.58 | -.43 | .77 |

*Note*. CFI=Comparative Fit Index; RMSEA=Root Mean Square Error of Approximation.

# SM7. The PSS-2&2 (German version)

Die folgenden Fragen beschäftigen sich mit Ihren Gedanken und Gefühlen während des letzten Monats. Bitte geben Sie für jede Frage an, wie oft sie in entsprechender Art und Weise gedacht oder gefühlt haben.

|  | nie | fast nie | manchmal | ziemlich oft | sehr oft |
| --- | --- | --- | --- | --- | --- |
| 1. Wie oft waren Sie im letzten Monat zuversichtlich, dass Sie fähig sind, Ihre persönlichen Probleme zu bewältigen? | 1 | 2 | 3 | 4 | 5 |
| 2. Wie oft hatten Sie im letzten Monat das Gefühl, dass sich die Dinge zu Ihren Gunsten entwickeln? | 1 | 2 | 3 | 4 | 5 |
| 3. Wie oft waren Sie im letzten Monat aufgewühlt, weil etwas unerwartet passiert ist? | 1 | 2 | 3 | 4 | 5 |
| 4. Wie oft haben Sie sich im letzten Monat über Dinge geärgert, über die Sie keine Kontrolle hatten? | 1 | 2 | 3 | 4 | 5 |

Auswertung

Skala Selbstwirksamkeit: Summe der Items 1 und 2

Skala Hilflosigkeit: Summe der Items 3 und 4

Deutsche Übersetzungen aus

Schneider, E. E., Schönfelder, S., Domke-Wolf, M., & Wessa, M. (2020). Measuring stress in clinical and nonclinical subjects using a German adaptation of the Perceived Stress Scale. *International Journal of Clinical and Health Psychology*, *20*(2), 173–181. https://doi.org/10.1016/j.ijchp.2020.03.004
